# Clinical and Molecular Characterization of *BSCL2* Mutations in a Taiwanese Cohort with Hereditary Neuropathy

**DOI:** 10.1371/journal.pone.0147677

**Published:** 2016-01-27

**Authors:** Cheng-Tsung Hsiao, Pei-Chien Tsai, Chou-Ching Lin, Yo-Tsen Liu, Yen-Hua Huang, Yi-Chu Liao, Han-Wei Huang, Kon-Ping Lin, Bing-Wen Soong, Yi-Chung Lee

**Affiliations:** 1 Department of Neurology, Taipei Veterans General Hospital, Taipei, Taiwan, ROC; 2 Department of Neurology, National Yang-Ming University School of Medicine, Taipei, Taiwan, ROC; 3 Brain Research Center, National Yang-Ming University, Taipei, Taiwan, ROC; 4 Department of Neurology, School of Medicine, National Cheng Kung University Hospital, Tainan, Taiwan, ROC; 5 Institute of Biomedical Informatics, National Yang-Ming University School of Medicine, Taipei, Taiwan; 6 Center for Systems and Synthetic Biology, National Yang-Ming University, Taipei, Taiwan; University of Florida, UNITED STATES

## Abstract

**Background:**

A small group of patients with inherited neuropathy that has been shown to be caused by mutations in the *BSCL2* gene. However, little information is available about the role of *BSCL2* mutations in inherited neuropathies in Taiwan.

**Methodology and Principal Findings:**

Utilizing targeted sequencing, 76 patients with molecularly unassigned Charcot-Marie-Tooth disease type 2 (CMT2) and 8 with distal hereditary motor neuropathy (dHMN), who were selected from 348 unrelated patients with inherited neuropathies, were screened for mutations in the coding regions of *BSCL2*. Two heterozygous *BSCL2* mutations, p.S90L and p.R96H, were identified, of which the p.R96H mutation is novel. The p.S90L was identified in a pedigree with CMT2 while the p.R96H was identified in a patient with apparently sporadic dHMN. *In vitro* studies demonstrated that the p.R96H mutation results in a remarkably low seipin expression and reduced cell viability.

**Conclusion:**

*BSCL2* mutations account for a small number of patients with inherited neuropathies in Taiwan. The p.R96H mutation is associated with dHMN. This study expands the molecular spectrum of *BSCL2* mutations and also emphasizes the pathogenic role of *BSCL2* mutations in molecularly unassigned hereditary neuropathies.

## Introduction

Mutations in the Berardinelli-Seip congenital lipodystrophy 2 gene (*BSCL2*) were originally identified in patients with autosomal recessive generalized congenital lipodystophy type 2 (MIM #269700) [1] and, later, were also found to cause a broad continuum of neurological diseases, including distal hereditary motor neuropathy (dHMN) type V (dHMNV; MIM #600794) [2,3,4], axonal form of Charcot-Marie-Tooth disease (CMT2) [4], Silver syndrome (MIM #270685) [2,3,4], pure or complicated hereditary spastic paraparesis [4] and, rarely, autosomal recessive progressive encephalopathy (MIM #615924) [5]. Silver syndrome is a rare form of hereditary spastic paraparesis in which spasticity of the legs is accompanied by amyotrophy of the hands and occasionally also the lower limbs [2,3]. *BSCL2* encodes seipin, which is an integral endoplasmic reticulum (ER) membrane protein containing a predicted structure of two transmembrane domains, a long luminal loop, and cytoplasmic N- and C-terminal tails [2,6]. Seipin is highly expressed in testis and nervous tissues, including brain and spinal cord [1]. Previous studies demonstrated that human seipin functions as a dodecamer [7] and plays an essential role in lipid droplet formation [8, 9], adipocyte differentiation [10–12], cellular triglyceride lipolysis [12], regulation of excitatory synaptic transmission [13, 14], and release of synaptic vesicles [14].

Seipinopathy is designated as a spectrum of neurologic diseases caused by *BSCL2* mutations [15]. Although a number of cases of seipinopathy have been reported worldwide, only three mutations in *BSCL2* were identified as cause for inherited neuropathy, including the p.N88S, p.S90L and p.S90W mutations [2–4, 16]. The p.N88S and p.S90L mutations were demonstrated to compromise protein folding of seipin, provoke endoplasmic reticulum (ER) stress, induce cell toxicity, and cause dHMNV, Silver syndrome, or CMT2 [15,17]. The p.S90W mutation was recently identified in a Korean pedigree with CMT2 by genome-wide linkage analysis and whole exome sequencing [16]. The small number of *BSCL2* mutations associated with inherited neuropathies implicates that the frequency of *BSCL2* mutations in patients with inherited neuropathies may be underestimated. This point is further supported by two recent studies which utilized whole exome sequencing to investigate the genetic cause of inherited neuropathies with earlier unsuccessful candidate gene testing and both identified mutations in *BSCL2* in their cases [18,19]. Currently, studies about seipinopathy in Han Chinese populations remain sparse, and *BSCL2* mutations have rarely been screened in inherited neuropathy cohorts before. The aim of this study is to investigate the frequency and spectrum of *BSCL2* mutations in a cohort of Taiwanese patients with genetically unassigned hereditary neuropathy. The clinical and molecular features of the identified *BSCL2* mutations were also characterized.

## Methods

### Patients

Eighty-four unrelated patients of Han-Chinese origin with inherited neuropathies of unknown genetic cause, including 76 with CMT2 and 8 with dHMN, were recruited into this study. These patients were chosen from a continuous series of 340 unrelated patients with CMT and 8 with dHMN recruited from the Neurology Clinics of Taipei Veterans General Hospital. Among these patients, CMT2 and dHMN were diagnosed in 103 and 8 patients, respectively, according to the guidelines described in the report of the 2nd Workshop of the European CMT consortium [20]. Among the CMT2 patients, 27 have been already proven as having a pathogenic mutation [21, 22, 23]. The remaining 76 CMT2 patients with unknown genetic cause and the 8 dHMN patients received mutational analyses of *BSCL2* in this study. A flow chart to clarify which patients were selected for sequencing analyses is available in the Supporting Information (S1 Fig). Peripheral blood samples were collected after obtaining the written informed consent from the patients or one of their parents for those younger than 18 years. The protocols for this study were approved by the Institutional Review Board of Taipei Veterans General Hospital. The individuals have provided written consent for the use of their information and videos as per the consent form for publication in a PLOS Journal.

### Mutation analyses

Genomic DNA was isolated from peripheral blood leukocytes. Mutational analyses of *BSCL2* was conducted by targeted sequencing in the same way as previously described [23]. In brief, a high-throughput targeted sequencing panel covering complete coding regions of *BSCL2* was designed to screen the patients for *BSCL2* mutations. This targeted sequencing panel also covers other 60 genes associated with inherited peripheral neuropathies (S1 Table). The enrichment of the targeted regions was performed using NimbleGen SeqCap EZ Choice Library system (Roche NimbleGen, Madison, WI). Enriched samples were sequenced on the HiSeq2000 platform (Illumina, San Diego, CA) using the paired-end 100bp protocol. All sequenced reads were mapped to the Human Genome version 19 (hg19/GRCh37). The BaseSpace pipeline (https://basespace.illumina.com/) and the Illumina VariantStudio software (http://variantstudio.software.illumina.com/) were used to variant calling and annotate variants, respectively. Sanger sequencing was performed to confirm the identified *BSCL2* variants. Amplicon sequences were aligned to the reference *BSCL2* coding sequence (NM_032667.6). The pathogenic property of the novel *BSCL2* variant was further assessed by *in silico* analysis with three programs, including Mutation Taster (http://www.mutationtaster.org) [24], PolyPhen-2 (http://genetics.bwh.harvard.edu) [25] and SIFT (http://http://sift.jcvi.org) [26]. The putative pathogenic variant was further validated in 500 neurologically healthy individuals of Han Chinese origin recruited at our hospital and the two large genetic polymorphisms databases, Exome Aggregation Consortium (ExAC, version 0.3; http://exac.broadinstitute.org) and dbSNP (Build 144; https://www.ncbi.nlm.nih.gov/snp). Phylogenetic conservation of the mutated amino acid residue was analyzed by aligning the amino-acid sequences of seipin orthologs from several species using the UniProt website (http://www.uniprot.org) [27].

### *In vitro* analyses of the functional significance of the *BSCL2* mutations

#### Plasmid constructs

The wild-type (WT) human seipin (*BSCL2*) expression plasmid (pCMV-SPORT6-BSCL2; BC093048) was purchased from transOMIC (Huntsville, AL). The mutations, c.269C>T (p.Ser90Leu) and c.287G>A (p.Arg96His), were introduced into the WT expression plasmids, separately, using QuikChange Site-Directed Mutagenesis kit (Stratagene, Santa Clara, CA).

#### Cell culture and transfection

HEK293 cells were maintained in Dulbecco’s modified Eagle’s medium (Gibco, Thermo Fisher Scientific, Waltham, MA) containing 10% fetal bovine serum and cultured in a humidified 5% CO_2_ incubator at 37°C. All transient transfections were performed using Lipofectamine 2000 (Invitrogen, Thermo Fisher Scientific).

#### Western blot analysis and protein solubility and stability assay

HEK293 cells were transfected with WT *BSCL2* or its mutant-expressing plasmids (p.S90L or p.R96H). Forty-eight hours post-transfection, cells were lysed in RIPA buffer supplemented with protease inhibitor cocktail (Merck Millipore, Darmstadt, Germany). Cell lysates were used for Western blotting and the protein concentration was determined using a Bradford protein assay kit (Bio-Rad, Hercules, CA).

To examine the protein solubility profile of seipin, sequential extractions were performed. Transfected cells were extracted with RIPA buffer and the cell lysates were centrifuged to generate the RIPA-soluble fraction. The resulting pellets were further re-solubilized with urea buffer (7M urea, 2M thiourea, 1% ASB-14, 40mM Tris, pH 8.5) to recover the most insoluble fraction. The protein from each fraction (40 μg) was separated on 8% SDS-PAGE, transferred to PVDF membranes, and immunoblotted with anti-BSCL2 antibodies (ab106793; Abcam, Cambridge, UK) followed by HRP-conjugated secondary antibodies. Detection was performed with a standard enhanced chemiluminescence assay (Perkin-Elmer Life Sciences, Boston, MA).

To determine the stability of the WT and mutant seipin proteins, cycloheximide-chase assays were conducted with cells transfected with different *BSCL2* constructs. Twenty-four hours after transfection, transfected cells were trypsinized and re-seeded into 6-well culture plates. After a further 24 hours, cycloheximide (CHX; Sigma-Aldrich, St. Louis, MO) was added to a final concentration of 0.1 mg/ml. Cell lysates were harvested at the indicated time points and subjected to Western blotting with the anti-BSCL2 antibody. Actin was used as a loading control. The ratios of seipin to actin were calculated densitometrically.

#### Real-time quantitative PCR (RT-qPCR) and ER stress detection

RT-qPCR was performed to measure the mRNA levels of *BSCL2* in cells transfected with different *BSCL2* constructs. Total RNA of transfected cells was harvested 24 hours after transfection and extracted using the Qiagen RNeasy mini kit (Qiagen, Valencia, CA). The cDNA synthesis was carried out with the SuperScript® III 1st Strand Synthesis Kit (Invitrogen). The RT-qPCR reactions were performed using a 7500 Fast Real-Time PCR System (Applied Biosystems, Thermo Fisher Scientific) in a 96-well format using the Fast SYBR® Green Master Mix (Applied Biosystems). The relative gene expression was normalized against *GAPDH* expression. To investigate the effect of the *BSCL2* mutations on ER stress, the mRNA expression levels of the ER stress biomarkers, BiP and CHOP, were examined in the transfected HEK293 cells by using RT-qPCR.

#### Immunofluorescence analyses

Forty-eight hours after transfection with the WT or mutant *BSCL2* constructs, cells were fixed with 4% paraformaldehyde and then permeabilized in 0.2% tween-20 (Sigma-Aldrich, St. Louis, MO). After blocking of non-specific binding with 5% bovine serum albumin, the cells were stained for seipin using the anti-BSCL2 antibody conjugated to Alexa 488 (Thermo Fisher Scientific) together with DAPI counter staining of cell nuclei. Immunofluorescent staining was examined under an Olympus FluoView FV10i confocal laser scanning fluorescence microscopy system with a 60X oil immersion objective (Olympus, Tokyo, Japan).

#### Cell viability assay

Cell counting kit-8 (CCK-8) colorimetric assay (Dojindo Molecular Technologies, Rockville, MD) was used to assess the cell viability. HEK293 cells (8 × 10^3^ cells per well) were grown in 96-well plates and transfected with different *BSCL2* constructs. Forty-eight hours post-transfection, CCK-8 solution (10 μl) was added to growing cultures and incubated at 37°C for 2 h. The absorbance at 450 nm was determined by Multiskan™ FC Microplate Photometer (Thermo Fisher Scientific). Cell viability was expressed as a percentage of that of the cells transfected with WT *BSCL2* expression plasmid.

## Results

### Identification of the *BSCL2* mutations

**Fig 1:**
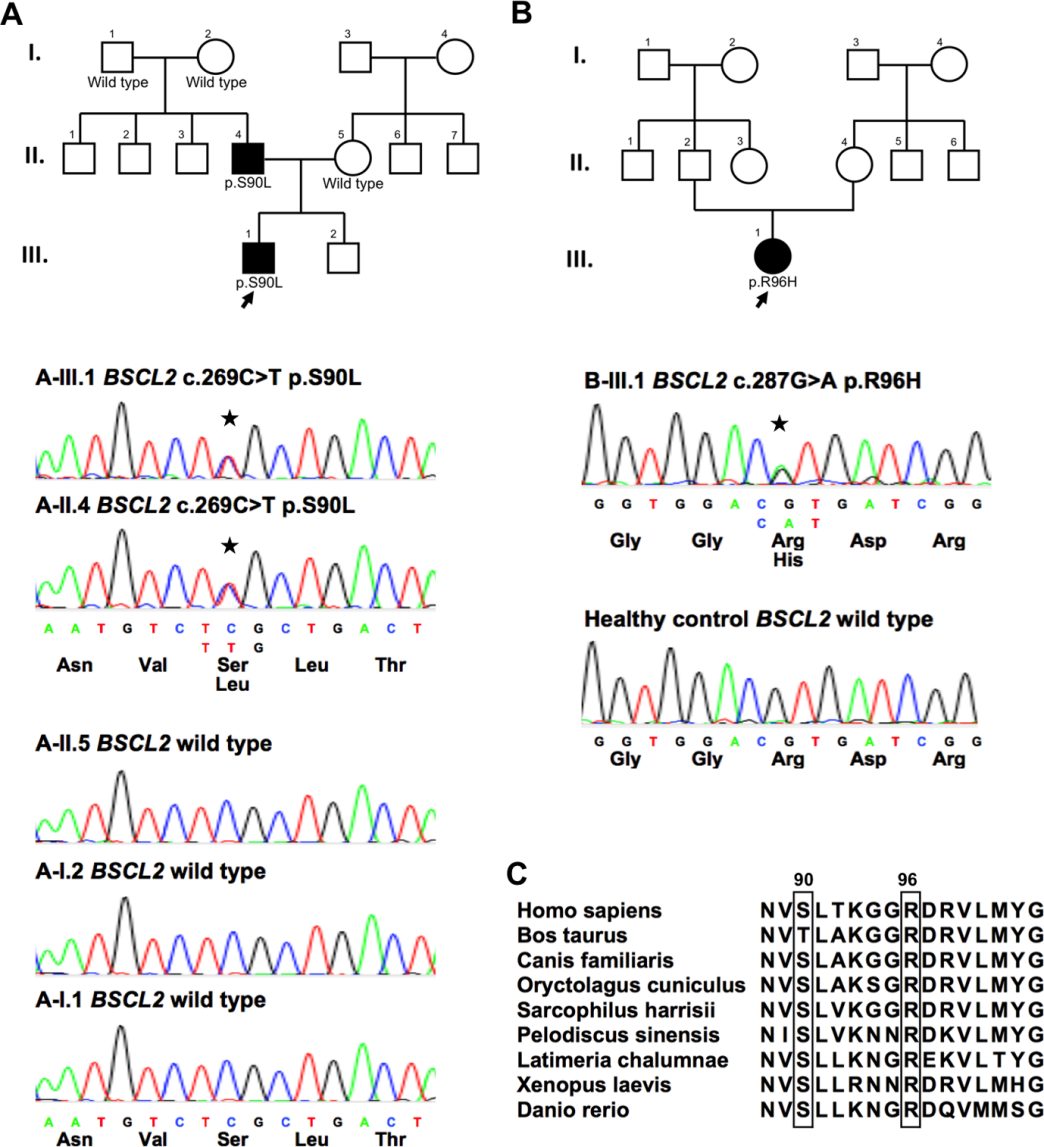
The pedigrees and sequencing data of the two families carrying *BSCL2* mutations. (A) The pedigree and the electropherograms of the individuals of the family carrying the *BSCL2* p.S90L (c.269C>T) mutation. The proband (III-1) is denoted by an arrow. Filled symbols represent affected members with neuropathy, open symbols indicate unaffected individuals, circles stand for female, and squares stand for male. (B) The pedigree and the electropherogram of the patient carrying the *BSCL2* p.R96H (c.287G>A) mutation. (C) The *BSCL2* p.S90L and p.R96H mutations reside in an evolutionarily conserved region, as shown by aligning the amino acid sequences of seipin protein orthologs from various species.

The *BSCL2*-targeted sequencing of the 84 patients with CMT2 or dHMN has an average coverage of 98.7% and read depth of 597X per targeted base. Two heterozygous missense variants in *BSCL2* were identified, including p.S90L (c.269C>T; ch11:62469965G>A) and p.R96H (c.287G>A; ch11:62469947C>T) (Fig 1A and 1B). The first variant, p.S90L, which was identified in a son and his father with CMT2, has been recognized as a disease-causative mutation in multiple previous reports [2,3,18]. The p.R96H is a novel *BSCL2* variant. It was identified in an apparently sporadic patient. Although the genomic DNAs of the patient’s families were unavailable, the p.R96H is not found in 500 ethnically matched healthy controls and is present in only one African out of the 60,067 individuals of diverse ethnicities in the databases of ExAC. The p.R96H is also absent in dbSNP database. The 90th and 96th amino acid residues of the human seipin protein are highly evolutionarily conserved (Fig 1C). The *BSCL2* p.R96H is also predicted as a pathogenic mutation by Mutation Taster, Polyphen-2 and SIFT programs (Table 1).

**Table 1:** Deleterious consequences of the *BSCL2* variants predicted by *in silico* analyses.

*BSCL2* Mutation	Changes in nucleotide	Changes in amino acid	Prediction programs			
			Mutation Taster	PolyPhen-2		SIFT
			Probability value	HumDiv	HumVar	SIFT score
Novel	c.287G>A	p.R96H	0.9993267	1	0.995	0.01 (Damaging)
Previously	c.263A>G	p.N88S	0.9999712	1	1	0.01 (Damaging)
reported mutations	c.269C>T	p.S90L	0.9999983	0.999	0.946	0.1 (Tolerated)
Remarks			All are predicted to be disease-causing	All are predicted as probably damaging		

### Clinical information of the patient carrying the *BSCL2* mutations

The clinical and electrophysiological features of the patients with *BSCL2* mutations in this study were summarized in Table 2. Patient A-III.1 is heterozygous for the *BSCL2* p.S90L mutation (Fig 1A). He had a slowly progressive distal muscle weakness and atrophy in the lower limbs since age 5 years and then in the hands since age 12. Neurological examination at age 23 revealed a pes cavus, generalized hypoactive deep tendon reflexes (DTRs) except for brisk knee jerks, symmetrical weakness and atrophy of the muscles in distal extremities, flexor plantar responses and a steppage gait (S1 Video). The Medical Research Council (MRC) scale scores were grade 4 for the intrinsic hand and feet muscles and dorsiflexors of his feet. Strength of other muscle groups was intact and all modalities of sensation were normal. The nerve conduction studies (NCS) demonstrated an axonal sensorimotor polyneuropathy (Table 1). His father, Patient A-II.4, also has a similar clinical phenotype since childhood and a *BSCL2* p.S90L mutation (Fig 1A). Neurological examinations at age 49 revealed a pes cavus, generalized areflexia except for brisk knee jerks, bilateral flexor plantar responses, symmetrical weakness and atrophy of the muscles in distal extremities, and a steppage gait with mild spastic feature (S2 Video). The MRC scale scores were grade 4 for finger extensor and flexor muscles and muscles in the legs, and grade 2–3 for the intrinsic hand and feet muscles. The NCS demonstrated an axonal sensorimotor polyneuropathy (Table 2). Patient B-III.1 is heterozygous for the *BSCL2* p.R96H mutation. She is the only child of her family and had an insidious onset and gradually progressive foot drop since age 10. Neurological examination at age 19 revealed a pes cavus, generalized hypoactive DTRs except for normal knee jerks, flexor plantar responses, weakness and atrophy of the muscles in the legs, and a steppage gait. The MRC scale scores were grade 2–3 for the dorsiflexors of her feet and toes, and grade 5 for her plantar flexor muscles. Strength of other muscle groups was intact and all modalities of sensation were normal. The NCS revealed a motor neuropathy without sensory involvement (Table 2).

**Table 2:** Clinical and electrophysiological features of the patients with *BSCL2* mutations.

	Family A (c.269C>T, p.S90L)		Family B (c.287G>A, p.R96H)
	A-III.1	A-II.4	B-III.1
Gender	Male	Male	Female
Age at onset (year)	5	10	10
Age at exam (year)	23	49	18
Clinical diagnosis	CMT2	CMT2	dHMN
Initial symptom	Unsteady gait	Unsteady gait	Unsteady gait
Atrophy and weakness	Distal UL + LL	Distal UL + LL	Distal LL
Knee jerks	Brisk	Brisk	Normal
Ankle jerks	Absent	Absent	Absent
Plantar response	Flexion	Flexion	Flexion
Gait	Steppage gait	Steppage gait with mild spastic feature	Steppage gait
Sensation test	Normal	Normal	Normal
Median nerve MNCV, m/s^a^	43.5	40.5	54.8
Median nerve CMAP, mV^a^	0.8	0.4	5.8
Peroneal nerve MNCV, m/s^a^	NR	NR	NR
Peroneal nerve CMAP, mV^a^	NR	NR	NR
Tibial nerve MNCV, m/s^a^	43.2	36.7	26.9
Tibial nerve CMAP, mV^a^	0.8	2.5	2.1
Sural nerve SNAP, μV^a^	2.6	3.3	14.7
Median nerve SNAP, μV^a^	38.3	19.0	52.9

CMT2: Charcot-Marie-Tooth disease type 2; dHMN: distal hereditary motor neuropathy; UL: upper limbs; LL: lower limbs; MNCV: motor nerve conduction velocity; CMAP: compound motor action potential; SNAP: sensory nerve action potential; NR: no response

^a^Normal values: median MNCV ≥ 51.9 m/s; median CMAP ≥ 6.4 mV; tibial MNCV ≥ 41.5 m/s; tibial CMAP ≥ 3.5 mV; sural SNAP ≥ 12 mV; median SNAP ≥ 17 mV.

### *In vitro* analyses of the functional significance of the *BSCL2* p.R96H mutations

**Fig 2:**
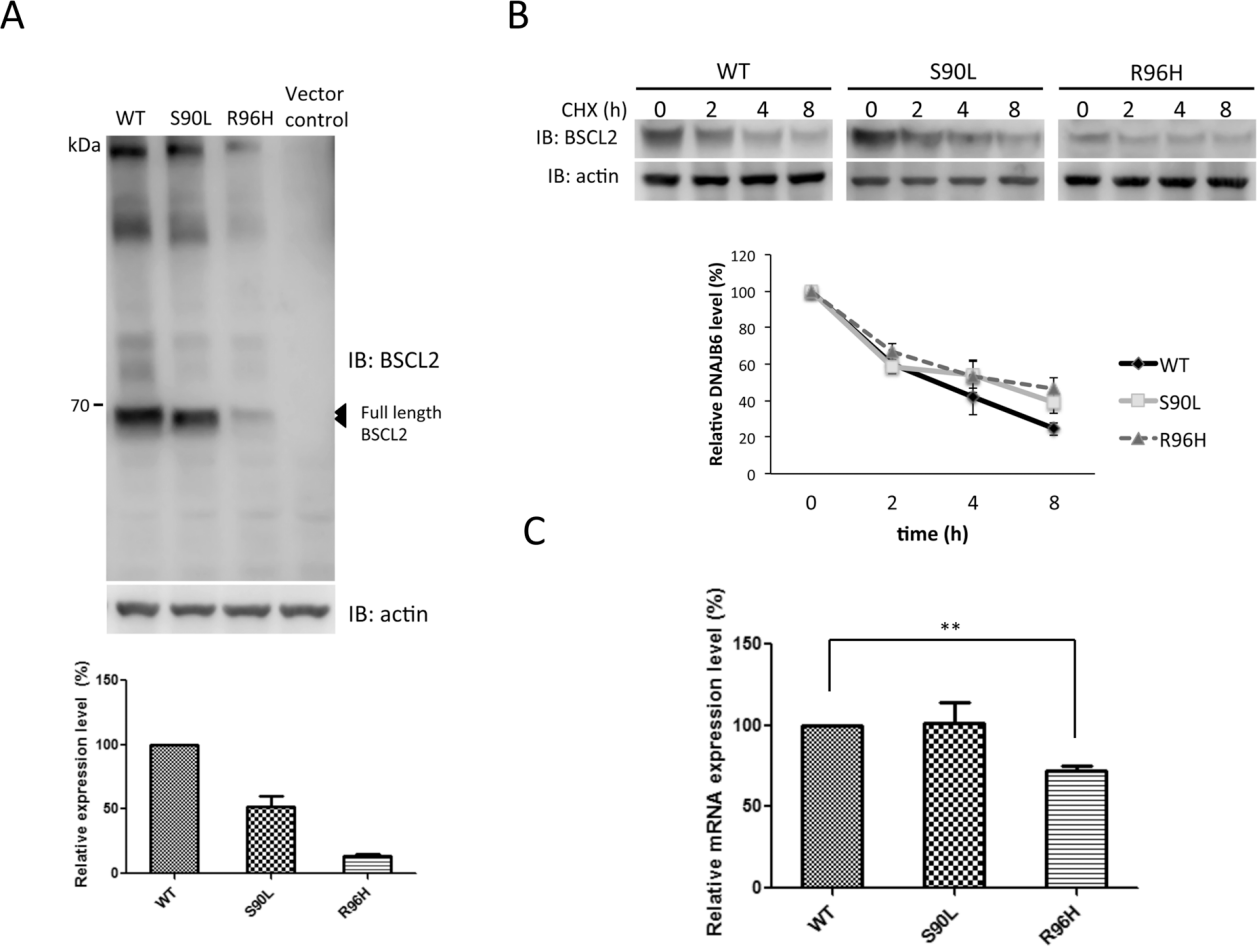
*In vitro* expression of *BSCL2* in HEK293 cells. (A) Representative western blot analysis of seipin in the HEK293 cells transfected with plasmids expressing wild-type (WT), S90L, or R96H seipin, or empty vector (vector control). Actin was used as a loading control. Densitometric quantification is shown below. The error bars indicate standard error of the mean (SEM) from 3 independent experiments. IB, immunoblotting. (B) HEK293 cells were transfected with expression plasmids for the WT, S90L, or R96H seipin for 48 hours and subsequently subjected to cycloheximide-chase assays. Representative western blots is shown above. All values are shown as means ± SEM (n = 4). (C) The messenger RNA (mRNA) levels of *BSCL2* in the HEK293 cells transfected with WT, S90L, or R96H seipin expression plasmids for 24 hours. The expression levels of *BSCL2* were normalized to those of GAPDH and expressed as a fraction of the WT samples, which was set as 100%. The error bars indicate SEM (n = 3). The asterisk indicates statistically significant difference (**, p < 0.01).

*BSCL2* p.S90L has been well characterized in previous *in vitro* studies and was used as a reference here [15, 17]. To investigate the impact of p.R96H mutation on the seipin function, we first utilized western blotting to evaluate whether the mutation influenced protein expression, and found that the steady-state level of the mutant R96H seipin was much lower than that of the WT protein (Fig 2A). Then, cycloheximide-chase assay was conducted and revealed that the p.R96H mutation did not compromise the seipin protein stability (Fig 2B). Further analysis of *BSCL2* mRNA levels in HEK293 cells transfected with different *BSCL2* constructs by RT-qPCR demonstrated that the steady-state mRNA level of p.R96H *BSCL2* was significantly lower than that of WT *BSCL2* (Fig 2C).

**Fig 3:**
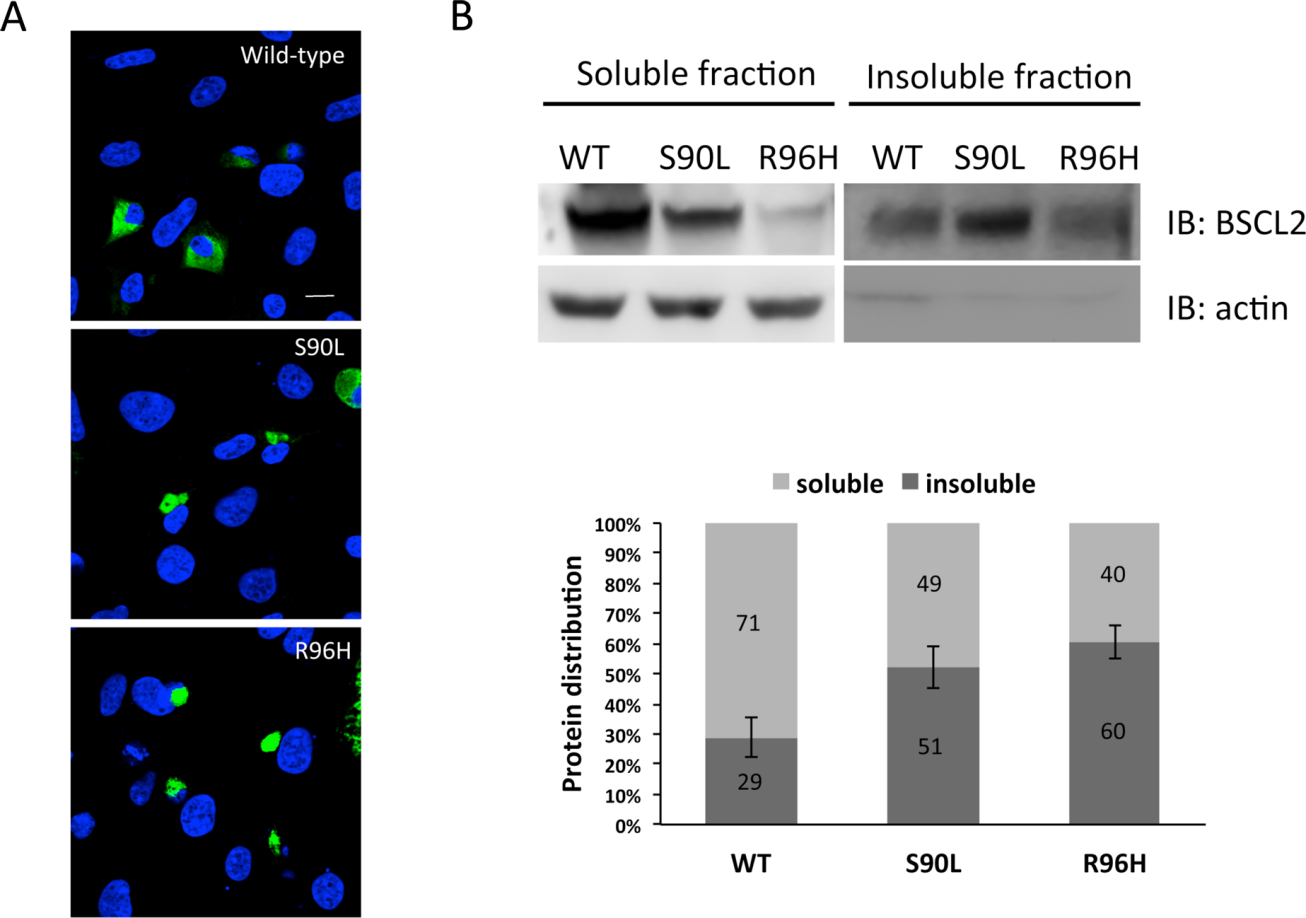
*In vitro* characterization of wild-type (WT) and mutant seipin proteins in HEK293 cells. (A) Representative images of immunofluorescence analyses of HEK293 cells transiently expressing WT, S90L, or R96H seipin labeled with anti-BSCL2 antibody (green) for staining seipin and 4’-6-diamidino-2-phenylindole (DAPI; blue) for nuclear counter stain. The images indicate that WT seipin was mainly expressed in the cytoplasm with a reticular staining pattern, whereas S90L or R96H mutants appeared to form large perinuclear aggregates. The experiments were repeated for three times and all had similar findings. (B) Images of representative immunoblots of the soluble and insoluble fractions from HEK293 cells transiently expressing WT, S90L, or R96H seipin. The levels of seipin in each fraction were measured by Western blot using the anti-BSCL2 antibody. The proportion of soluble and insoluble parts of each sample calculated by densitometric analyses is shown below. The error bars indicate SEM from 3 independent experiments. IB, immunoblotting.

Since both N88S and S90L mutants were shown to form intracellular inclusions, which were nearly undetectable in cells expressing the WT seipin [17], it was interesting to define the intracellular features of the mutant R96H seipin. We performed immunofluorescence analysis to visualize the intracellular patterns of WT or the mutant seipin proteins in HEK293 cells. Cells expressing WT seipin showed a seipin-specific reticular staining throughout the cytoplasm, whereas cells expressing the S90L or R96H mutants demonstrated large cytoplasmic aggregates with seipin-specific staining (Fig 3A).

Because the *BSCL2* p.R96H mutation increases the aggregation tendency of seipin, we next examined the biochemical solubility of WT seipin and the seipin mutants expressed in HEK293 cells. The cells transfected with different *BSCL2* constructs were extracted with RIPA buffer (soluble fraction) first and subsequently extracted with urea buffer to recover insoluble seipin proteins. Western blot analysis revealed that more than half of the S90L and R96H mutant proteins were found in the insoluble fraction of cell lysates, whereas the WT protein was detected predominantly in the soluble fraction (Fig 3B). These results indicate an obvious tendency of the S90L and R96H mutant seipin proteins to form detergent-insoluble aggregates, leading to a significant depletion of soluble seipin in the cytosol.

**Fig 4:**
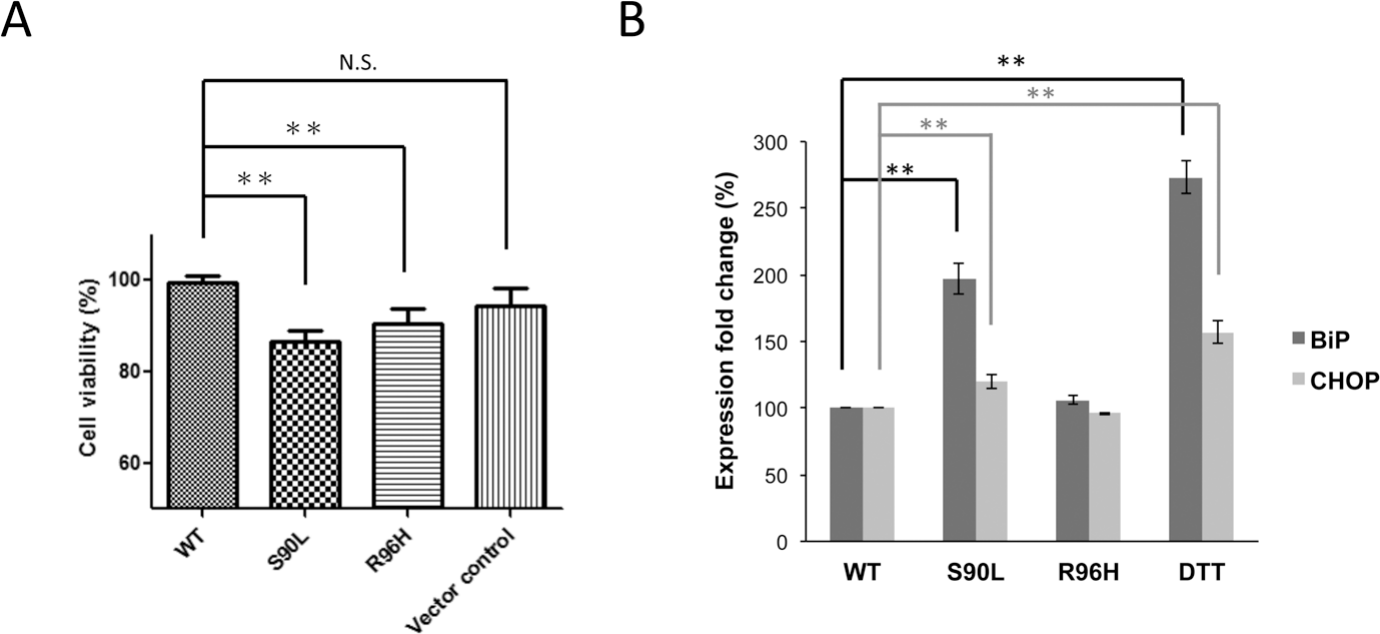
Cell viability and endoplasmic reticulum (ER) stress assays. (A) The Cell counting kit-8 (CCK-8) colorimetric assay revealed that expression of S90L or R96H seipin led to a significant reduction in viability of HEK293 cells. The percentage of the viable cells in HEK293 cells transfected to express wild-type seipin was set as 100% and used for comparison. Values are shown as means ± SEM of 24 independent transfections (**, p < 0.01). (B) The activation of ER stress was evaluated by quantitative real-time PCR (qRT-PCR), measuring the expression levels of *BIP* and *CHOP* mRNA. Cells exposed to DTT or transfected with S90L seipin expression plasmid represented the positive controls. All values (mean ± SEM, n = 4) were normalized to *GAPDH* mRNA levels (**, p < 0.01). Cells expressing R96H seipin did not have a significantly higher expression levels of *BIP* or *CHOP* mRNA compared with cells expressing WT seipin, suggesting that the R96H seipin did not induce ER stress.

To determine whether expression of R96H seipin causes cell toxicity, HEK293 cells were transfected with the WT or mutant *BSCL2* constructs for 48 hours and then assessed the cell viability by CCK-8 colorimetric assay. As shown in Fig 4A, compared with WT seipin, both S90L and R96H seipin have a significant cellular toxicity. Moreover, we also investigated the effect of R96H seipin on the ER stress because both N88S and S90L mutants were reported to induce unfolded protein response and ER stress [17]. We analyzed the mRNA expression of two ER stress markers, BiP and CHOP, in HEK293 cells transfected with the WT or mutant *BSCL2* constructs for 48 hours by RT-qPCR. HEK293 cells exposed to an ER stress stimulator dithiothreitol (DTT) or transfected to express S90L seipin were used as positive controls. As shown in Fig 4B, cells expressing R96H seipin did not have a significantly higher expression level of Bip or CHOP as compared with cells expressing WT seipin, indicating that the R96H seipin did not induce ER stress.

## Discussion

In this study, we identified two heterozygous missense mutations in the *BSCL2* gene after screening 84 unrelated patients with molecularly-unassigned CMT2 or dHMN for *BSCL2* mutations. One is p.S90L in a family with CMT2, and the other is p.R96H in an apparently sporadic patient with dHMN. The p.S90L has been well documented as pathogenic before [2,3,18], whereas the p.R96H is a novel mutation. The pathogenicity of the *BSCL2* p.R96H mutation is supported by the following facts. First, the *BSCL2* p.R96H mutation is not present in the 1,000 control chromosomes from 500 neurologically healthy Taiwanese individuals. Second, the mutation is absent in the large genetic polymorphisms databases, dbSNP, and is present with an extremely rare allele frequency (1/ 120,134) in the ExAC database. Third, the *BSCL2* p.R96H mutation occurs at an evolutionarily conserved amino acid residue of the human seipin and multiple computational predictive programs, including Mutation Taster, Polyphen-2 and SIFT, support its deleterious effect. Four, *in vitro* functional studies demonstrated that the p.R96H mutation results in a low protein expression level, increases the aggregation tendency of seipin, and decreases cellular viability without increasing ER stress. Following the *BSCL2* p.N88S, p.S90L and p.S90W mutations [2,3,16], the p.R96H mutation is the fourth *BSCL2* mutation identified to cause inherited neuropathy.

Because the first two seipinopathy-related mutations, *BSCL2* p.N88S and p.S90L, were originally identified in patients with dHMN or Silver syndrome [2,3], seipinopathy is commonly recognized as a spectrum of motor neuron disorders presenting with variable combination of upper and lower motor neuron signs. However, CMT2 was also found in a minor portion of patients with seipinopathy later. In a study of 90 patients from three families with the p.N88S mutation, 20% of the patients had CMT2 [4]. To date, the p.S90W mutation was only found in patients with CMT2 [16]. In our study, the p.S90L mutation was also identified in a father and son with CMT2. These findings emphasize the importance of considering the role of peripheral sensory involvement in seipinopathy and also underlie the contribution of *BSCL2* mutations to CMT2.

Although the *BSCL2* p.R96H mutation results in dHMN, a typical phenotype of seipinopathy, its molecular pathogenic mechanisms seem to be different from those of the two most common seipinopathy-related mutations, *BSCL2* p.N88S and p.S90L mutations. Previous studies have shown that the p.N88S and p.S90L mutations enhance ubiquitination and degradation of seipin by the ubiquitin–proteasome system (UPS) and appear to induce seipin proteins misfolding [17]. These misfolded mutant proteins accumulate in the ER and then provoke ER stress leading to cell toxicity [17]. This study has demonstrated that the p.R96H mutation results in a profoundly decreased level of soluble seipin protein due to decreased *BSCL2* mRNA expression and increased propensity to form insoluble aggregates. Expression of the R96H mutant seipin also reduced cellular viability without provoking ER stress. The discrepancies between the molecular pathogenic mechanisms of different mutations within the same gene are not rare in inherited neuropathy. For example, different mutations in *GJB1* or *MPZ* may result in disparate intracellular trafficking patterns of the GJB1 or P_0_ proteins, although they all cause CMT [28,29].

The prevalence of *BSCL2* mutations in inherited neuropathy seems to be low. In previous studies, Murphy et al. identified *BSCL2* mutations in one out of 425 patients with CMT and another one patient out of 61 HMN patients in their inherited neuropathy clinic [30]. Fridman et al. found that five out of 1,652 patients with inherited neuropathy had *BSCL2* mutations (0.3%; 5/1652) [31]. Our study demonstrated that the frequency of *BSCL2* mutations in Taiwanese patients with inherited neuropathy is low (0.57%; 2/348). Pooling the data from the above three studies shows that the frequency of *BSCL2* mutations in patients with inherited neuropathy is approximately 0.36% (9/2486), indicating that *BSCL2* mutations are an uncommon cause of inherited neuropathy.

In conclusion, *BSCL2* mutations account for a small number of patients with inherited neuropathies in Taiwan. The *BSCL2* p.R96H mutation is a novel cause of dHMN. *In vitro* functional study revealed that the p.R96H mutation results in a remarkably low seipin expression and reduced cellular viability. This study expands the molecular spectrum of *BSCL2* mutations and also emphasizes the pathogenic role of *BSCL2* mutations in molecularly unassigned hereditary neuropathies.

## Supporting Information

S1 Fig The flow chart demonstrating how to select patients for *BSCL2* analysis in the study. (DOCX)

S1 Table The list of the genes covered by the targeted sequencing panel in the study. (DOCX)

S1 Video Patient A-III.1 presented steppage gait. (MP4)

S2 Video Patient A-II.4 presented steppage gait with mild spastic feature. (MP4)
