# Supplementary material for: Clinical and Molecular Characterization of BSCL2 Mutations in a Taiwanese Cohort with Hereditary Neuropathy
Source: PLoS One. 2016 Jan 27;11(1):e0147677. doi: 10.1371/journal.pone.0147677 (PMC4729478; doi:10.1371/journal.pone.0147677)
Supplement: S1 Fig — (DOCX) [file pone.0147677.s001.docx]

**Figure S1.** The flow chart demonstrating how to select patients for *BSCL2* analysis in the study. CMT: Charcot-Marie-Tooth disease; CMT2: axonal CMT; HMN: hereditary motor neuropathy.

**76 CMT2 patients and 8 HMN patients received mutational analyses of *BSCL2***

***BSCL2* p.R96H in one HMN patients**

***BSCL2* p.S90L in one CMT2 patient**

**Excluding patients with clear genetic diagnosis**

**Selecting patients with CMT2 or HMN for mutational analyses of *BSCL2***

**The cohort of patients with inherited neuropathy**

**Mutational analyses of *BSCL2* by targeted next-generation sequencing**

**76 CMT2 patients and 8 HMN patients with unknown molecular diagnoses**

**103 CMT2 patients and 8 HMN patients**

**348 unrelated patients with inherited neuropathy (340 CMT patients and 8 HMN patients)**
