# Supplementary material for: Clinical and Molecular Characterization of BSCL2 Mutations in a Taiwanese Cohort with Hereditary Neuropathy
Source: PLoS One. 2016 Jan 27;11(1):e0147677. doi: 10.1371/journal.pone.0147677 (PMC4729478; doi:10.1371/journal.pone.0147677)
Supplement: S1 Table — (DOCX) [file pone.0147677.s002.docx]

**Table S1. The list of the genes covered by the targeted sequencing panel in the study**

| *AARS* | *HSPB1* | *PLEKHG5* |
| --- | --- | --- |
| *AIFM1* | *HSPB3* | *PMP22* |
| *ATP7A* | *HSPB8* | *PRPS1* |
| *BICD2* | *IGHMBP2* | *PRX* |
| *BSCL2* | *INF2* | *RAB7* |
| *CTDP1* | *KARS* | *REEP1* |
| *DCTN1* | *KIF5A* | *SBF1* |
| *DHTKD1* | *LITAF* | *SBF2 (MTMR13)* |
| *DNAJB2 (HSJ1)* | *LMNA* | *SETX* |
| *DNM2* | *LRSAM1* | *SH3TC2* |
| *DYNC1H1* | *MARS* | *SLC5A7* |
| *EGR2* | *MED25* | *SOX10* |
| *FBLN5* | *MFN2* | *SPTLC1* |
| *FGD4* | *MPZ* | *SPTLC2* |
| *FIG4* | *MT-ATP6* | *SURF1* |
| *GARS* | *MTMR2* | *TFG* |
| *GDAP1* | *MYH14* | *TRIM2* |
| *GNB4* | *NDRG1* | *TRPV4* |
| *HARS* | *NEFL* | *TTR* |
| *HINT1* | *PDK3* | *YARS* |
| *HK1* |  |  |
